# The Book World of Medicine and Science

**Published:** 1900-02-24

**Authors:** 


### THE BOOK WORLD OF MEDICINE AND SCIENCE.

HERMANN LUDWIG FERDINAND VON HELMHOLTZ. By JOHN GRAY M'KENDRICK, M.D., LL.D., F.R.S.S., L. and E. (London: T. Fisher Unwin. 1899. Price 3s. 6d.)

A LIFE of Helmoltz such as this could only be written by a man thoroughly accomplished in all that concerns the physicist and the physiologist, for a good deal of its charm is due not merely to the interest which must always attach to the life history of one whose investigations and whose theories made such an impression on his contemporaries as was the case with Helmholtz, but depends also, in no small degree, upon the fact that the presentment which it offers of the scientific doctrines involved is itself the work of a master. A life of Helmholtz without a running comment on the sciences around which his whole career revolved would be almost without point or meaning. As it is, the book before us is almost as interesting as a record of scientific progress as it is as a life of a great investigator in the realms of physics and physiology. Helmholtz, who was born at Potsdam, near Berlin, on August 31st, 1821, was of mixed nationality so far as descent was concerned, for while his father was a German, his mother was the daughter of a Hanoverian artillery officer of the name of Penne, a lineal descendant of William Penn, the great Quaker, who founded Pennsylvania, and his grandmother on his mother's side sprang from a family of French refugees of the name of Sauvage. Thus Helmholtz had German, English, and French blood in his veins. For the first seven years of his life he was a weakly boy, confined for long periods to his room, and frequently to his bed; but he showed much activity of mind, and his parents gave up to him much of their time and attention. At an early age he read largely, and when at length he went to school his mind quickly opened up. Already at eight years of age he was able, it is said, to astonish his teachers by his knowledge of many fundamental truths in geometry. As he grew stronger and became able to ramble with his father in the beautiful country about Potsdam he began to look at nature with his own eyes, and thus commenced that inquiry into and that study of natural phenomena which form the striking features of his life. As Helmholtz approached manhood it was his desire to devote his life to the study of physics, but his father's means were too limited to allow of his son taking up a career in which it would have been next to impossible to earn a livelihood, and he wisely counselled his son to study medicine in the first instance. Thus it happened that he was entered as a bursar at the Royal Medico-Chirurgical Friedrich Wilhelm Institute in Berlin, an academy for the education of youths of promise, given freely on the condition that they afterwards became surgeons in the Prussian army. In due time, then, he obtained his diploma and became an army surgeon. Even before his qualification, however, he had begun his career of original research, and in his inaugural thesis, which he presented at the age of twenty-one, he made an important contribution to minute anatomy in regard to the relation between the nerve fibre and the nerve cell in the ganglia of the nervous system. From this date, namely, 1842, when his first paper was published, down to the year of his death in 1894, when he had reached the age of seventy-three, papers flowed from his pen in almost uninterrupted succession. With the exception of one year, 1849, he published at least one important paper, and usually three or four each year, so that when his life's work was over no fewer than 217 distinct papers and books represented his labours. In one sense a life of such constant labour lends itself badly to the biographer, for amid such steady occupation but little time is left for those incidents by which a biography is rendered interesting. On the other hand to a writer who knows how to make the most of his subject there is in such a life an unending supply of interesting material, and if in this case the life of Hermann von Helmholtz turns out to be in large degree a history of the progress of those sciences to the development of which his life was devoted, the work is none the less true as a biography, for his life lay in his work, and the record of his discoveries really gives the reader the fullest conception possible of how he lived, and what he did, and what he aimed at.

UNWIN'S CHAP BOOK, 1899-1900. (London: T. Fisher Unwin. Price 1s.)

NOT as a preface but as part of the title on the opening page we have this legend in which the virtues of the book are sung:—

"This Chapbook is a Book for Chaps,
And women too, and girls (perhaps).
This Chapbook's not a 'homely tract,'
To make you go to sleep; in fact,
This Chapbook staves off idle naps,
For it a Town of Books was sacred."

Out of this it would be possible to raise many diverse and generally incorrect notions as to the character of its contents. The book, in fact, is a rather interesting collection of biographical, historical, descriptive, and other details, often taking the "interview" form, regarding the people who have written the books published by T. Fisher Unwin, of London. Still, we all like to know about the authors whose works we enjoy, and undoubtedly the stories here told are told very well. Whether, indeed, "a town was sacked" for its production, or only Mr. Unwin's own book warehouse, we cannot say, but the book is interesting, and the illustrations are admirable. One can almost forget that it is an advertisement; and this is high praise.

A CONTRIBUTION TO THE SURGERY OF FRACTURES AND DISLOCATIONS OF THE UPPER EXTREMITY. By T. E. PLATT, M.S., F.R.C.S. (London: H. K. Lewis. Pp. 228. Illustrated. Price 10s.)

MR. PLATT, who has lately been resident surgical officer of the Manchester Royal Infirmary, has based this extremely praiseworthy monograph upon an analysis of some seven hundred consecutive cases observed by him in that capacity. The plan of the work is naturally dictated by the subject, which affords ample opportunity for tabular statement and systematic treatment. Mr. Platt's undoubted experience certainly justifies his occasional difference with, and criticism of, the authors he so freely quotes, but we feel that the value of his book chiefly appertains to the very full and careful notes he took with such admirable persistency during his term of office. Not the least interesting portions of the work are those which relate to treatment, and we notice with pleasure the free use which Mr. Platt has made of Skiagraphy. Indeed, the book is illustrated by no less than a score of reproductions of exceedingly beautiful skiagrams. We heartily congratulate the author on the powers that have led him to the satisfactory accomplishment of his task, and we have little doubt that his volume will enjoy the honour of consultation by every writer on systematic surgery.

